# Effects of home-based cardiac rehabilitation integrated in the cardiac care bridge transitional care program on the physical functioning of older patients who are frail: secondary analysis of a randomized trial

**DOI:** 10.1093/ptj/pzag020

**Published:** 2026-03-06

**Authors:** Michel S Terbraak, Lotte Verweij, Patricia Jepma, Marike van der Schaaf, Harald T Jørstad, Ron J G Peters, Bianca M Buurman, Wilma J M Scholte op Reimer

**Affiliations:** Location Academic Medical Center, Department of Cardiology, Amsterdam UMC, Meibergdreef 9, 1105AZ, Amsterdam, Noord Holland, the Netherlands; Center of Expertise Urban Vitality, Faculty Health, Amsterdam University of Applied Sciences, Amsterdam 1097 DZ, Noord Holland, the Netherlands; Institute for Implementation Science in Health Care, Department of clinical nursing, University of Zurich, Universitätstrasse 84, Zurich 8006, Zurich, Switzerland; University Hospital Zurich, Department Center for Clinical Nursing Science, Rämistrasse 100, Zurich 8091, Zurich, Switzerland; Location VU University Medical Center, Department of Medicine for Older People, Amsterdam UMC, de Boelelaan, Amsterdam 1109, Noord Holland, the Netherlands; Amsterdam Public Health, Department Aging & Later Life, Van der Boechorststraat 7, 1081 BT, Amsterdam, Noord Holland, the Netherlands; Center of Expertise Urban Vitality, Faculty Health, Amsterdam University of Applied Sciences, Amsterdam 1097 DZ, Noord Holland, the Netherlands; Amsterdam UMC, Location University of Amsterdam, Department Rehabilitation, Meibergdreef 9, 1105AZ, Amsterdam, Noord Holland, the Netherlands; Amsterdam Movement Sciences, Department Ageing and Vitality, De Boelelaan 1117, 1081 HZ, Amsterdam, Noord Holland, the Netherlands; Location Academic Medical Center, Department of Cardiology, Amsterdam UMC, Meibergdreef 9, 1105AZ, Amsterdam, Noord Holland, the Netherlands; Amsterdam Movement Sciences, Department Ageing and Vitality, De Boelelaan 1117, 1081 HZ, Amsterdam, Noord Holland, the Netherlands; Location Academic Medical Center, Department of Cardiology, Amsterdam UMC, Meibergdreef 9, 1105AZ, Amsterdam, Noord Holland, the Netherlands; Location VU University Medical Center, Department of Medicine for Older People, Amsterdam UMC, de Boelelaan, Amsterdam 1109, Noord Holland, the Netherlands; Amsterdam Public Health, Department Aging & Later Life, Van der Boechorststraat 7, 1081 BT, Amsterdam, Noord Holland, the Netherlands; Amsterdam UMC, Location University of Amsterdam, Department Internal Medicine, Section of Geriatric Medicine, Meibergdreef 9, 1105AZ, Amsterdam, Noord Holland, the Netherlands; Location Academic Medical Center, Department of Cardiology, Amsterdam UMC, Meibergdreef 9, 1105AZ, Amsterdam, Noord Holland, the Netherlands; HU University of Applied Sciences Utrecht, Department Research Group Chronic Diseases, Heidelberglaan 7, 3584 CS, Utrecht, Utrecht, the Netherlands

**Keywords:** patients, physical, older, cardiac rehabilitation, home-based, frail

## Abstract

**Importance:**

Older patients hospitalized for cardiovascular disease (CVD) are at risk of physical function decline and adverse health outcomes. Cardiac rehabilitation (CR) improves physical functioning but is underutilized by older patients. Home-based CR potentially improves utilization, yet its effectiveness in older patients who are frail remains understudied.

**Objective:**

The objective of this study was to investigate the effects of a transitional-care integrated home-based CR program on physical functioning in older patients who are frail after CVD hospitalization.

**Design:**

This was a prespecified secondary analysis of physical functioning at the 6-month follow-up in the cardiac care bridge multicenter randomized trial.

**Setting:**

A home-based setting was used.

**Participants:**

The study participants were patients who were frail and ≥70 years old after CVD hospitalization.

**Intervention:**

The intervention was transitional care followed by physical therapist led home-based CR and community nurse visits.

**Main outcomes and measures:**

The primary physical function outcome was the Short Physical Performance Battery (SPPB) in cases with complete follow-up data. Secondary outcomes included the 2-min step test, grip strength, and Amsterdam Linear Disability Scale. Sensitivity analyses included an intention-to-treat analysis by multiple imputation of the full cohort.

**Results:**

In total, 85 of 153 participants in the intervention group and 85 of 153 participants in the control group were analyzed (mean age = 82.6 [SD = 6.3] years; 46% men; median of 2 [interquartile range = 1–4] comorbidities). At the 6-month follow-up, more participants in the intervention group than in the control group demonstrated SPPB improvement (61% vs 51%) or maintenance (29% vs 12%), and fewer deteriorated (11% vs 37%). The mean SPPB values at 6 months were 6.3 (SD = 0.3) and 5.5 (SD = 0.2), respectively, with a mean difference of 0.8 (95% CI = 0.0-1.6), favoring the intervention group. No between-group differences were observed in the 2-min step test, grip strength, or Amsterdam Linear Disability Scale.

**Conclusions:**

Among older patients who were frail and had CVD, a comprehensive transitional-care program with integrated home-based CR resulted in clinically relevant improvements in physical functioning.

**Relevance:**

The results substantiate the effectiveness of home-based CR in older patients who are frail and have CVD.

## Introduction

Older patients hospitalized for cardiovascular disease (CVD) are at high risk of physical function decline.^1^ This risk is particularly high in older patients with a history of previous hospitalization or with signs of frailty, such as malnutrition, an increased risk of falls, and impaired cognitive functioning.^2^ Decline in physical function is furthermore associated with major health problems, such as loss of independence and quality of life.^3^

Numerous studies have demonstrated that older patients who are not frail, have cardiac issues, and participate in cardiac rehabilitation (CR) demonstrate improvements in physical functioning.^4–12^ In contrast, the effect of CR in older patients who are frail is unknown because of their limited participation in CR trials. Furthermore, central barriers to CR participation comprise transportation challenges and comorbidity-related issues in this vulnerable group of patients.^13^ In recognition of this knowledge gap, the American Heart Association and European Society of Cardiology have issued a call to action for the development of innovative CR delivery strategies, including home-based CR.^14^

A novel approach in the delivery of CR involves the integration of home-based CR within a transitional-care program. Transitional-care interventions commonly use a case management strategy, including a comprehensive geriatric assessment and delivering a wide range of interventions tailored to address the diverse needs of patients.^15–18^ In contrast to traditional CR, this approach has 2 advantages: first, it focuses not only on cardiac disease but also on geriatric conditions that may limit daily activities and recovery, and second, transitional care has the potential to reduce adverse health outcomes (eg, by reducing nonadherence to pharmacological and exercise treatment).^19^^,^^20^ We developed the cardiac care bridge (CCB) program, which was a transitional-care program designed specifically for older patients who are frail and hospitalized for cardiac issues and included case and disease management with the integration of home-based CR.^21^ The primary aim of the program, as previously reported,^21^^,^^22^ was to reduce hospital readmissions and mortality. This was not achieved. Other studies have explored related outcomes, including patient and caregiver experiences, medication adherence (showing no effect overall but modest improvements among those not using a multidose drug dispensing system), adherence to home-based CR, and cost-effectiveness (indicating no differences in health care costs but lower quality-adjusted life years because of higher involvement of informal caregivers). To date, the program's effects on anxiety, depression, and physical functioning remain unexamined. This paper addresses this gap by focusing on the effect of the CCB program on physical functioning. We hypothesized that the CCB home-based CR intervention would improve patients’ physical function at 6 months.

## Methods

### Cardiac care bridge design

We performed a prespecified secondary analysis focusing on physical functioning within the CCB trial.^21^ Detailed information on the CCB trial design, content of home-based CR, and primary outcome results, have been published elsewhere.^21–23^ In brief, the CCB was a single-blind multicenter randomized controlled trial performed across 6 medical centers in the Netherlands with subsequent home-based follow-up (June 2017 to March 2020). Patients were randomized to either usual care (control group) or the CCB transitional-care program (intervention group), which included home-based CR. The study was approved by the Medical Ethics Committee of the Amsterdam University Medical Centre (Protocol ID: MEC2016_024) and registered in the Dutch Trial Register (NTR6316; April 6, 2017). Informed consent from all enrolled patients was obtained within 72 hours of hospital admission.

### Usual care

**Figure 1:**
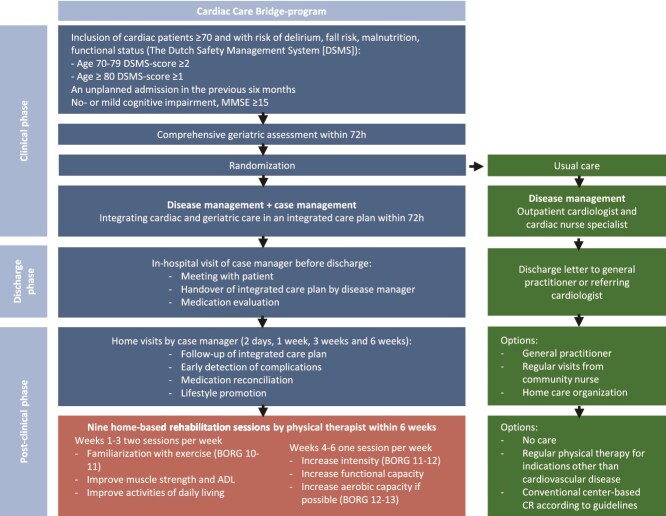
Overview of the Cardiac Care Bridge Program Including Home-Based Cardiac Rehabilitation (CR). Blue depicts nurse-coordinated care, the middle lowest box (red) shows the exercise components of home-based cardiac rehabilitation provided by physical therapists, and boxes on the right side (green) depict usual care. In addition, physical therapists provided coaching on an active lifestyle and monitored complications. Abbreviations: ADL = activities of daily living; BORG = Borg scale of perceived exertion; DSMS = Dutch safety management system; MMSE = Mini-mental state examination. Adapted with permission from Terbraak et al,^23^ licensed under CC BY-NC-ND 4.0.

Usual care included routine care in the hospital and after discharge, including a comprehensive geriatric assessment, and outpatient cardiologist and cardiac nurse specialists. Usual care also included the possibility for referral to conventional center-based CR according to current guidelines.^24–26^ Conventional CR comprises a 6- to 8-week multidisciplinary program addressing cardiovascular risk factors and psychosocial support and including exercise sessions twice weekly. In addition, participants were allowed to follow their regular physical therapist treatment for indications other than CVD (Figure 1).

### Intervention

Patients randomized to the intervention group received the CCB transitional-care program (Figure 1) during hospitalization, at hospital discharge, and after discharge, consisting of 3 components: case management, disease management, and home-based CR.^21^^,^^22^ The cardiac research nurse collaborated with the community nurse and physical therapist to arrange post-discharge care. The community nurse acted as case manager and visited the participant and cardiac research nurse in hospital for a handover of the integrated care plan, which included relevant medical information. In the post-discharge phase, the community nurse scheduled visits at 2 days and 1, 3, and 6 weeks after discharge. During these visits, the community nurse reviewed the integrated care plan and performed disease management by assessing health status, medication use, and potential medication related problems. A medication check was performed jointly with the pharmacist during the first visit. Physical therapists providing home-based CR were trained to improve their knowledge of geriatric syndromes and exercise options in older patients with CVD, and to improve interprofessional collaboration during a joint training with the cardiac research nurses and community nurses. Physical therapists concentrated on enabling patients to engage in daily activities in line with their personal goals, while promoting an active lifestyle and conducting regular exercises to improve strength, physical functioning, and endurance if feasible.^23^ The exercise protocol has been described in detail elsewhere.^23^ The first home visit by the physical therapists was a joint intake with a community nurse and the participant to discuss goals and desired activities (Figure 1). Physical therapists were instructed to provide 2 weekly home-based CR sessions in weeks 1 to 3 and 1 in weeks 4 to 6 after discharge, with a maximum of 9 sessions.^21–23^^,^^27^ However, the number and timing of sessions could be adapted to the patient’s needs, allowing both shortening and extension of the treatment period at the therapist’s discretion. No structured contact was scheduled between 6 weeks and 6 months, but participants were advised to continue their home exercise program independently. Adherence to these exercises was supported through self-management strategies practiced during the CR sessions. Home-based CR was provided according to the Dutch and European CR guidelines.^24–26^^,^^28^ Community nurses reviewed the integrated care-plan, health status, and medication, promoted an active lifestyle, and monitored whether patients followed the exercise advice communicated by the physical therapist through a logbook kept at the patients’ homes.^21^^,^^22^

### Study population

Patients included in the CCB trial were ≥70 years old, hospitalized at a cardiology or cardiothoracic surgery department, and at high risk of functional loss, defined as Dutch Safety Management System (DSMS) screening instrument^29^ score indicating a high risk of functional loss or hospital admission in the 6 months prior to the current admission. The DSMS includes 4 domains on frailty: risk of impaired daily functioning, delirium, falls, and malnutrition. DSMS scores range from 0 to 4. Patients ≥80 years old were considered at high risk if their DSMS score was ≥1, whereas those 70 to <80 years old were deemed at high risk if their DSMS score was ≥2.

Exclusion criteria were an inability to provide consent or follow instructions because of severe cognitive impairment (Mini-Mental State Examination score of <15) or delirium; a life expectancy of ≤3 months (as assessed by a physician); congenital heart disease; transfer from or discharge to a nursing home; and planned discharge to another hospital (department) not participating in the CCB trial.

### Outcomes

Our primary outcome was physical functioning at the 6-month follow-up, measured with the Short Physical Performance Battery (SPPB). This time point enables the assessment of late intervention effects (eg, follow-up exercise advice by the community nurses) and facilitates meaningful cross-study comparisons.^17^^,^^30^^,^^31^

Secondary outcomes at the 6-month follow-up included exercise capacity assessed with the 2-minute step test (TMST),^32^^,^^33^ grip strength measured using the Jamar dynamometer,^34^ and participants’ self-perceived daily activity levels measured with the Amsterdam Linear Disability Scale (ALDS).^35^^,^^36^

### Data collection

A physical therapist (M.S.T.) trained 4 cardiac research nurses to perform the SPPB, grip strength measurement, and TMST according to standard operating procedures. Within 72 hours after admission, cardiac research nurses extracted baseline characteristics (age, sex, left ventricular ejection fraction) and performed the Mini-Mental State Examination,^37^ Charlson Comorbidity Index,^38^ and ALDS assessments. SPPB, TMST, and grip strength were measured before discharge. At the 6-month follow-up, nurses assessed SPPB, TMST, grip strength, and ALDS in patients' homes.^21^

The SPPB is a standardized measure of lower extremity function, scored from 0 to 12, with lower scores indicating greater dysfunction.^39–41^ It is associated with the risk of losing physical independence (0-3 = high risk, 4-9 = medium risk, 10-12 = low risk).^29^^,^^38^^,^^40–44^ At group level, a change of 0.5 or more is considered clinically meaningful.^42^

Exercise capacity was assessed using the TMST, a valid and reliable test for older populations, suitable for home settings.^32^^,^^33^ Participants stepped in place for 2 min, lifting their knees to a height midway between the patella and iliac crest, starting with the right leg. The total number of right leg lifts to the set height was recorded. Grip strength was measured using the Jamar dynamometer (Lafayette Instrument Co).^34^ Each hand was tested 3 times, and the highest score from either hand was recorded as the maximum grip strength.

The ALDS is a 77-item bank assessing daily activities, hierarchically ordered from simple to complex based on item response theory.^35^^,^^36^ In our study, 15 items were selected, tailored to participants' activity levels, ranging from “putting on a t-shirt” to “climbing stairs with groceries.” Responses were "I can" or "I cannot" perform the activity, with "not applicable" recorded if the task was irrelevant or unknown. Scores range from 0 to 100, with lower scores indicating greater disability.

### Statistical methods

Baseline characteristics are presented as means and SDs for normally distributed variables, as median and interquartile ranges (IQRs) for nonnormally distributed variables, and as frequencies and percentages for categorical variables.

We presented individual change from baseline to the 6-month follow-up in waterfall plots of the primary outcome (SPPB). As the SPPB can only be scored in integers, at the individual level, a change of 1 or more points on the SPPB was deemed clinically relevant.^43^ Each participant was categorized into 1 of 3 groups: deterioration (≤ − 1), no change (=0), or improvement (≥1) in physical functioning. We performed a post hoc chi-square analysis to evaluate distribution differences between the intervention and control groups. The effect of the intervention was analyzed using an analysis of covariance by performing a linear model with the SPPB 6-month follow-up score as the dependent variable. Independent variables included group allocation (intervention vs control), baseline SPPB score, age, and sex. The intervention effect was interpreted based on the regression coefficient for the group variable, along with its 95% CI and *P*-value. Statistical assumptions included linearity, independence, and normality of residuals, which were visually assessed using residual and Q-Q plots. We performed a complete case analysis, including only participants with an SPPB score at the 6-month follow-up. Secondary outcome analyses for the TMST, grip strength, and ALDS were performed using the same approach as for the primary analysis, adjusted for their respective baseline values, age, and sex.

In an exploratory sensitivity analysis, we introduced additional predefined factors as covariates and interaction terms to the regression model, to assess potential effect modification on or confounding of the primary outcome. We explored the interactions for study site and covariates selected for their established association with physical function recovery. These included baseline comorbidity levels, socioeconomic status (based on education, and living alone vs with a partner), and age groups (70-79 years vs ≥80 years). To assess the association between intervention dose and response (SPPB score at 6 months), we conducted subgroup analyses comparing participants who attended at least 50% and 75% of the 9 intervention sessions with all controls. To assess the impact of missing data on the primary outcome, additional sensitivity analyses were conducted using multiple-imputation techniques in all 306 participants in the CCB trial. We used the Multivariate Imputation by Chained Equations (MICE) package in R.^44^ MICE performs multiple imputations by iteratively modeling each variable with missing values using the other variables in the dataset. We performed multiple imputations to create 5 imputed datasets. The MICE algorithm was run for 10 iterations to ensure adequate convergence of the imputation models. The multiple imputation utilized estimates based on baseline age, sex, study group, SPPB baseline score, and factors related to missingness, such as DSMS baseline score, comorbidity level, and ALDS baseline score. The primary outcome analysis was subsequently repeated in the imputed dataset.

To assess potential selection bias due to missing outcome data, we compared baseline characteristics of participants with and without 6-month SPPB data separately for the intervention and control groups. We used an independent *t* test and chi-square test for, respectively, continuous normally distributed and nonnormally distributed or categorical variables.

All statistical analyses were performed using R (version 4.1.2; R Foundation for Statistical Computing, Vienna, Austria) and R-Studio 2022.07.0. A *P*-value of <.05 was considered statistically significant.

## Results

**Figure 2:**
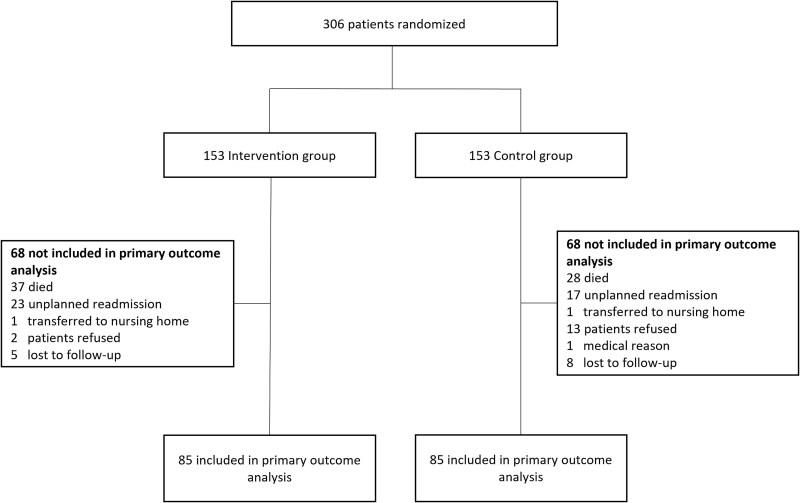
Flowchart of this Study.

Complete 6-month SPPB data were available for 170 of the 306 patients (55.6%) in the CCB trial (Figure 2). As expected in this population of older people who were frail, missing physical functioning data were largely due to mortality, hospital readmissions, refusal of follow-up measurements, or loss to follow-up (eg, relocation to a care facility).

Participants with available SPPB data at the 6-month follow-up in the intervention and control groups had mean ages of 82.5 (SD = 5.8) and 82.7 (SD = 6.7) years, respectively; 46% and 55%, respectively, were men; and 51% and 42%, respectively, were living together with a partner (Table 1). Participants in both groups had a median of 2 comorbidities. There were no statistically significant differences between the intervention and control groups except for the length of hospital stay: 6 (IQR = 4–9) and 8 (IQR = 5–11) days, respectively (*P* = .007). Physical functioning scores were numerically but statistically not significantly lower in the intervention group than in the control group (SPPB scores = 4 [IQR = 1–7] and 5 [IQR = 3–7]; *P* = .108).

**Table 1:** Baseline characteristics of cases with complete primary outcome (SPPB) data at 6 months*^a^*

Characteristic or Score	Intervention Group (*n* = 85)	Control Group (*n* = 85)
Characteristic		
Age, y, mean (SD)	82.5 (5.8)	82.7 (6.7)
Sex, men	39 (46)	47 (55)
Level of education*^b^*		
Primary	41 (48)	31 (36)
Secondary	22 (26)	23 (27)
Higher	22 (26)	30 (35)
Living together	43 (51)	36 (42)
Length of stay, d, median (IQR)	6 (4-9)	8 (5-11)*^c^*
LVEF, %, mean (SD)	36.8 (12.4)	37.3 (14.1)
Charlson Comorbidity Index, median (IQR)	2 (1-4)	2 (1-4)
MMSE, mean (SD)	25.5 (3.4)	25.1 (3.4)
Fear of falling, median (IQR)*^d^*	2 (0-6)	2 (0-6)
Baseline score for physical functioning		
SPPB	*n* = 66, 19 missing	*n* = 65, 20 missing
Mean (SD)	4.1 (3.2)	5.1 (3.1)
0-4: severe	35 (53)	29 (45)
5 or 6: moderate	13 (20)	17 (26)
7 or 8: mild	11 (17)	12 (19)
9-12: no limitation	7 (11)	7 (11)
Steps	*n* = 51, 34 missing	*n* = 49, 36 missing
Median (IQR)	29 (13-44)	34 (20-55)
Grip strength, mean (SD)		
Men	28.1 (10.1)	28.3 (7.7)
Women	17.8 (5.4)	19.1 (14.4)
ALDS score, median (IQR)	77 (60-89)	78 (69-89)

^a^ Data are reported as numbers (percentages) of participants unless otherwise indicated. Measurements were performed <72 h after hospital admission, except for the Short Physical Performance Battery (SPPB), 2-min step test, and grip strength test, which were mainly conducted shortly before discharge. At baseline, 19 intervention group and 20 control group participants were unable or unwilling to rise from bed, in which case the SPPB was not feasible; in addition, 34 and 36 participants in the respective groups declined to perform the 2-min step test, citing concerns regarding its physical demands. These participants were discharged from the hospital before a revisit could take place. Abbreviations: ALDS = Amsterdam Linear Disability Scale; IQR = interquartile range; LVEF = left ventricle ejection fraction; MMSE = Mini-Mental State Examination.

^b^ Primary education: = elementary or primary school; secondary education = prevocational, senior general, or preuniversity; higher education = higher professional or university.

^c^ *P* < .05.

^d^ Measured with numeric rating scale scored from 0 (no fear) to 10 (maximal fear).

### Outcomes

**Figure 3:**
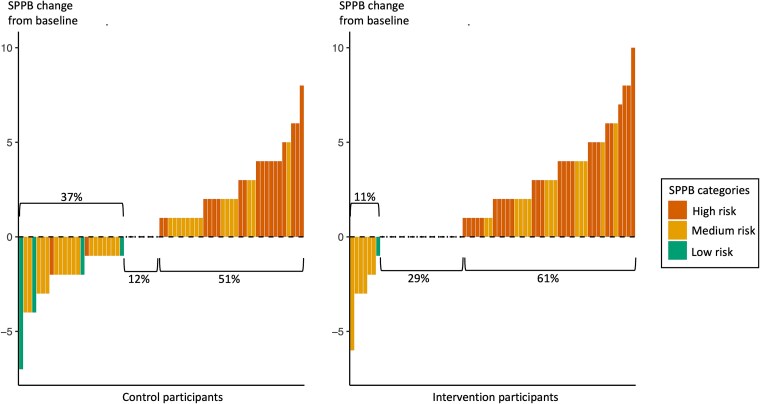
Waterfall Plot of Change in the Short Physical Performance Battery (SPPB) from Baseline to the 6-month Follow-up for the Intervention and Control Groups. SPPB categories: baseline risk of losing physical independence (0-3 = high risk, 4-9 = medium risk, 10-12 = low risk); χ^2^ analysis of SPPB change direction (deterioration [≤ − 1], no change [=0], improvement [≥1]) between groups was statistically significant (*P* = .001), favoring the intervention.

At the 6-month follow-up, improvement of physical functioning as measured by the SPPB was seen in 61% of the intervention group participants and 51% of the control group participants, maintenance was seen in 29% and 12%, and deterioration was seen in 11% and 37%, respectively (Figure 3). A chi-square test indicated a statistically significant difference in the distribution of SPPB change categories (*P* = .001). No patients in the intervention group with a severe baseline risk of deterioration in physical functioning demonstrated further deterioration.

All conditions for the linear regression models were met. The adjusted mean SPPB values at the 6-month follow-up in the intervention group and the control group were 6.3 (SD = 0.3) and 5.5 (SD = 0.2), respectively, with a mean difference of 0.8 (95% CI = 0.0-1.6, *P* = .049) (Table 2), above the predefined threshold of a clinically meaningful group difference of 0.5 SPPB point. For the secondary outcomes at 6 months, there were no statistically significant between-group differences (Table 2).

**Table 2:** Primary and secondary outcomes at baseline and at 6 months in cases with complete data*^a^*

Outcome, No. (%) of Participants	Baseline	6-mo Follow-up		Mean Difference: Adjusted
		Unadjusted	Adjusted	
Primary: SPPB score*^b^*				
Intervention, 66 (78)	4.1 (3.2)	6.1 (3.2)	6.3 (5.7 to 6.8)	0.8 (0.0 to 1.6)*^c^*
Control, 65 (76)	5.1 (3.1)	6.1 (2.6)	5.5 (4.9 to 6.0)	
Secondary				
2-min step test				
Intervention, 51 (60)	33.2 (27.8)	42.0 (29.9)	44.2 (36.9 to 51.5)	3.9 (−6.7 to 14.5)
Control, 49 (58)	37.7 (25.7)	45.9 (29.5)	40.3 (32.6 to 47.9)	
Handgrip strength, kg, in men				
Intervention, 39 (100)	28.1 (10.1)	25.4 (10.4)	25.6 (23.5 to 27.7)	−0.8 (−3.7 to 2.0)
Control, 46 (98)	28.3 (7.7)	27.0 (8.3)	26.4 (24.2 to 28.3)	
Handgrip strength, kg, in women				
Intervention, 45 (98)	17.8 (5.4)	15.2 (6.1)	14.6 (12.8 to16.4)	0.9 (−1.8 to 3.5)
Control, 35 (92)	19.1 (14.4)	14.0 (5.0)	13.8 (11.8 to 15.6)	
ALDS				
Intervention, 85 (100)	71.3 (18.0)	70.9 (17.7)	72.4 (69.4 to 75.4)	−1.8 (−6.1 to 2.5)
Control, 85 (100)	76.1 (12.6)	75.8 (16.8)	74.2 (71.2 to 77.2)	

^a^ Numbers and percentages of participants were based on 85 intervention group and 85 control group participants with complete Short Physical Performance Battery (SPPB) follow-up data. Unadjusted outcome scores are presented as means (SDs); adjusted (for baseline, age, and sex) scores are presented as least squares means (95% CIs). The effect sizes for the outcomes are shown as between-group differences in the least squares mean change (95% CIs). Abbreviation: ALDS = Amsterdam Linear Disability Scale.

^b^ Total scores on the SPPB range from 0 to 12, with lower scores indicating more severe physical dysfunction; each component (the standing balance test, the gait-speed test [as assessed by a 4-m walk], and the strength test [as assessed by the time needed to rise from a chair 5 times]) is scored on a scale from 0 to 4.

^c^ *P* < .05.

Introduction of the covariates and interaction analyses of study site (*P* > .342), comorbidity levels (*P* = .470), educational level (*P* > .054), living status (*P* = .787), and age groups (70–79 years vs ≥80 years; *P* = .255) identified no statistically significant (*P* < .05) effect modification of the primary outcome or evidence of confounding (change intercept of >10%).

In the intervention group, the median time from hospital discharge to the first physical therapist session was 8 days (IQR = 5-16), and the median treatment duration (interval between first and last sessions) was 37 days (IQR = 20-54). The final session occurred at a median of 49 days (IQR = 30-69) after discharge, indicating that most programs lasted ~6 to 7 weeks. The median number of sessions was 4 (IQR = 2-6). Ten participants (10%) completed 8 or 9 sessions within the first 6 weeks. Between 6 weeks and 6 months, 29 participants (30%) received additional sessions (median = 2 [IQR = 1-3]).

Of the 153 patients in the control group, 6 (4%) participated in center-based CR, of whom 4 (5%) were included in the subsample with complete follow-up SPPB data (*n* = 85). In this subsample, 30 control group patients (35%) reported receiving physical therapy during the first 3 months after discharge, compared to 53 intervention group patients (62%) who received physical therapy as part of the program. From 3 to 6 months after discharge, 21 control group patients (25%) received physical therapy, versus 31 patients (36%) in the intervention group, with medians of 8 and 4 sessions, respectively. Of 85 intervention group patients, 24 (28%) received only community nurse visits without any home-based CR sessions by the physical therapists, 19 (22%) received fewer than 5 (<50%) home-based CR sessions, 39 (46%) participated in 5 or more sessions, and 33 (39%) participated in 7 (>75%) or more sessions. Reasons for receiving fewer than 5 sessions were as follows: patient not motivated (*n* = 30), already receiving physical therapy with a noncardiac focus (*n* = 7), or unspecified reason (*n* = 6). No association was found between the intervention dose (<50%, >50%, or > 75%) and the SPPB score at 6 months.

In the multiple-imputation datasets of all 306 participants, sensitivity analyses (linear model adjusted for baseline SPPB, age, sex, DSMS, ALDS, and comorbidity) showed a similar trend (mean SPPB difference = 0.5 [95% CI = −0.2 to 1.2]; *P* = .158) as the complete case analysis but did not reach statistical significance for the primary outcome (Suppl. Table 1). Incorporating incompleteness as both a covariate and an interaction term in the linear model resulted in a shift of the intercept exceeding 50%.

In the intervention group, compared to participants with complete data, those missing 6-month SPPB data (*n* = 68, 44%) were less likely to live together with a partner (34% vs 51%; *P* = .049), and women showed a weaker grip strength (15.5 vs 17.9; *P* = .048) (Suppl. Table 2). In the control group, compared to participants with complete data, those missing 6-month follow-up data (*n* = 68, 44%) had lower initial SPPB scores (3 vs 5; *P* = .018) and achieved fewer steps in the 2-min test (22 vs 34; *P* = .001), and women had a weaker grip strength (17.9 vs 19.3; *P* = .048).

## Discussion

This study investigated the effects of home-based CR integrated in the CCB program on physical functioning of older patients who are frail and have CVD. More participants in the intervention group maintained or improved physical function, with fewer showing deterioration compared to the control group. In our primary analysis, statistically significant and clinically meaningful improvements in physical function (SPPB) were observed at 6 months in the intervention group compared to usual care. However, this was not confirmed in the multiple-imputation sensitivity analysis, and no significant differences were observed between groups for endurance (TMST), grip strength, or self-reported physical disability (ALDS).

The positive effect on physical functioning cannot be attributed solely to home-based CR, as no dose–response association was found between the number of sessions and SPPB follow-up scores. However, the statistical power for this analysis was limited, which may explain the absence of a significant association. Additionally, the combined intervention, including community nurses and their follow-up on physical therapy exercise advice, likely contributed to the intervention's effectiveness.

In the intervention group, a higher proportion of participants received physical therapy during the intervention and follow-up period, including home-based CR sessions. However, the total number of sessions was comparable or even higher in the control group during the later phase (median of 8 vs 4 sessions). The content of these sessions also differed substantially. Physical therapy in the intervention group was embedded within home-based CR and focused on goal-setting, physical activity coaching, and self-management, whereas physical therapy in the control group was generally directed at comorbid musculoskeletal problems and relied more on passive treatment modalities. Therefore, although differences in exposure to physical therapy could have contributed to the observed improvement in SPPB scores, it is more plausible that the content and behavioral focus of the intervention explain the between-group differences rather than the amount of physical therapy received.

Because of known floor and ceiling effects of the SPPB,^45–48^ patients with extreme scores tend to either maintain their current level of function or regress to the mean. Those with low initial scores are more likely to improve, whereas those with high scores may decline. We observed no improvements in participants who had low-risk SPPB scores at baseline, whereas from these participants 4 in the control group and 1 in the intervention group deteriorated. This substantiates the possible ceiling effect of the SPPB. Most participants however had baseline SPPB scores in the medium- to high-risk range for losing physical independence, indicating vulnerability. Although the intervention group had slightly higher baseline risk scores (not statistically significant), the clinically meaningful 0.8-point difference (95% CI = 0.0-1.6) in SPPB outcomes could be crucial for maintaining independence at home^41^^,^^42^^,^^49–52^; however, studies with longer follow-up periods are needed to substantiate this.

We found no significant differences between the intervention and control groups in self-reported daily activity disability (ALDS), endurance (TMST), or grip strength. High ALDS scores suggest a potential ceiling effect, possibly because of overreporting and the insensitivity of the selected items.^36^^,^^53^^,^^54^ Both groups showed limited endurance, with low TMST step counts, indicating daily activities may be as intense as aerobic exercise for both groups. Grip strength showed a normal decline with age, and no differences were observed between the groups,^55^^,^^56^ likely because of the lack of nutritional support in the intervention and the focus of the intervention on improvement of daily physical functioning instead of handgrip specifically.

Our study strengthens the evidence for the effectiveness of home-based CR integrated in transitional care on physical functioning,^17^ extending it to older patients who are frail, a typically understudied population. With an average age of 82 years and baseline SPPB scores of ≤5, our participants were more frail than those in previous studies (average age = 73 years; SPPB score = 6).^5^ The between-group SPPB difference of 0.8, which is statistically significant in the primary analysis, aligns with findings from other studies on physical activity in older patients.^5^^,^^50–52^^,^^57^ More patients in the intervention group improved or maintained physical function, whereas fewer experienced deterioration, highlighting the potential of home-based CR integrated in the CCB program to prevent functional decline in this population. However, as previously reported, this potential effect was not substantiated in our trial, as the CCB program did not reduce hospital readmissions or mortality^22^ and was not cost-effective within 1 year of follow-up.^58^ A longer follow-up period, such as 2 years or more, may be needed to fully assess cost-effectiveness.^59^

### Strengths and limitations

There are several strengths to our study. First, its randomized controlled trial design ensures a robust evaluation of the intervention. Second, our focus on older patients who were frail and had CVD enabled the generalization of the effectiveness of home-based CR on physical function in this understudied population.^30^^,^^39^^,^^41^^,^^50–52^^,^^60^ Finally, our study builds on previous research that demonstrated the feasibility of home-based CR integrated in transitional care in older patients who are frail, showing that it can be effectively implemented^23^ when some flexibility is allowed in adapting the program to patients’ individual needs and capabilities.

Some aspects of our study warrant consideration. First, the inclusion of numerous patients who either died or were readmitted to the hospital before starting home-based CR suggests we included individuals who were extremely frail and might have required alternative treatments, such as palliative care. To assess the impact of missing data, we conducted multiple-imputation analyses. Although the imputed results were consistent with the complete case findings, the primary SPPB outcome lost statistical significance. Further analysis revealed that accounting for missingness as a covariate or interaction significantly affected the intercept, indicating that participants with missing data at 6 months experienced a different intervention effect.

We furthermore observed no significant interactions between missing variables and the primary outcome in the sensitivity analyses. However, compared to complete cases, those missing 6-month SPPB data were more likely to live alone, had lower SPPB baseline scores, lower step counts, and female patients weaker grip strength, potentially indicating a higher risk of frailty and physical function decline.^61–63^ As a result, our findings may not be generalizable to populations of people who are even more frail and may require alternative approaches rather than rehabilitation (eg, palliative care). Differentiating between older patients who are frail and can benefit from home-based CR and those needing different care remains a challenge.

Another limitation concerns the moderate intervention fidelity. Approximately half of the participants in the intervention group received fewer than 5 of 9 planned home-based CR sessions within 6 weeks of discharge. Physical therapists often chose to distribute the planned sessions over a longer period to enable ongoing coaching on physical activity and self-management rather than focusing solely on exercise delivery within a fixed 6-week period, or to stop treatment earlier once patients’ physical activity goals were achieved. This flexible approach was consistent with the intervention’s person-centered design and the heterogeneity of recovery trajectories in frail older adults. Therefore, the observed variation in the number of sessions reflects both patient-level barriers, such as limited motivation or different care needs, and professional adaptation to individual capacities and goals. These findings highlight the tension between protocol adherence and individualized care in real-world settings and point to the need for adaptive delivery models that preserve both fidelity and flexibility.^64^

Finally, as this study was a secondary analysis of the main trial, power calculations for physical functioning measurements were not performed, which may have resulted in underpowered outcomes and the potential for type 2 errors, leading to an underestimation of differences between the intervention and control groups.

### Future perspectives

We observed improvements in physical functioning from the home-based CR integrated in the CCB program, although Jepma et al. found no effect on rehospitalizations or mortality.^22^ Although it differed from traditional CR, elements such as enhancing confidence in physical activity potentially contributed to improving physical functioning. However, aerobic training was often not feasible because of physical limitations or a focus on first improving physical function. Therefore, integrating aspects of both home-based cardiac (eg, exercise safety, monitoring cardiac decompensation) and geriatric rehabilitation may be the most suitable approach for this population.

Our study successfully included frail older patients hospitalized for CVD, allowing us to deliver and evaluate home-based CR in this understudied population. However, process evaluations revealed that patients preferred their usual health care providers over those in the CCB program.^65^ Therefore, educating and training existing health care providers to deliver home-based CR and motivate frail older patients to participate, could improve patient participation.

## Conclusions

A comprehensive transitional-care program with integrated home-based CR may improve aspects of physical functioning in frail older patients with cardiac conditions, although the effect was not consistent across all analyses and functional measures*.* Future research may focus on optimizing the intervention by tailoring individual exercise parameters.

## Supplementary Material

PTJ-2025-0105_R2__Suppl_Material_pe_pzag020

## Data Availability

On request via email to m.s.terbraak@hva.nl.

## Clinical trial registration

The study was registered in the Dutch Trial Register (NTR6316; April 6, 2017).
