# Supplementary material for: Effects of home-based cardiac rehabilitation integrated in the cardiac care bridge transitional care program on the physical functioning of older patients who are frail: secondary analysis of a randomized trial
Source: Phys Ther. 2026 Mar 6;106(4):pzag020. doi: 10.1093/ptj/pzag020 (PMC13064644; doi:10.1093/ptj/pzag020)
Supplement: PTJ-2025-0105_R2__Suppl_Material_pe_pzag020 [file ptj-2025-0105_r2__suppl_material_pe_pzag020.pdf]

Supplementary Table 1. Baseline characteristics complete cases primary outcome SPPB 6 months vs non-complete cases

	COMPLETE CASES 6 MONTH		MISSING SPPB 6 MONTH	
**Characteristics**	**Intervention (n=85)**	**Control (n=85)**	**Intervention (n=68)**	**Control (n=68)**
Age	82.5 (±5.8)	82.7 (±6.7)	82.4 (±6.5)	81.9 (±6.2)
Sex (male)%	39 (46)	47 (55)	31 (46)	39 (57)
Level of education[a] n(%)				
Primary	41 (48)	31 (36)	25 (37)	30 (44)
Secondary	22 (26)	23 (27)	30 (44)	21 (31)
Higher	22 (26)	30 (35)	13 (19)	17 (25)
Living together n(%)	43 (51)	36 (42)	23 (34)*	32 (47)
Length of stay, days, median (IQR)	6 (4-9)	8 (5-11)	6 (4-8)	8 (5-13)
LVEF %	36.8 (±12.4)	37.3 (±14.1)	38.3 (±11.2)	32.5 (±14.6)
Charlson, median (IQR)	2 (1-4)	2 (1-4)	3 (1-4)	3 (2-4)
MMSE	25.5 (±3.4)	25.1 (±3.4)	24.6 (±3.6)	23.9 (±4.0)
Fear of falling[b]	2 (0-6)	2 (0-6)	3 (0-5)	3 (0-5)
**Baseline scores physical functioning**				
SPPB median (IQR)	4 (1-7)	5 (3-7)	3 (2-5)	3 (0-6)*
Steps median (IQR)	29 (13-44)	34 (20-55)	26 (0-37)	22 (0-37)*
Gripstrength				
Men	28.2 (±10.2)	28.3 (±7.7)	25.2 (±6.6)	27.6 (±7.0)
Women	17.9 (±5.5)	19.3 (±14.6)	15.5 (±4.5)*	17.2 (±11.3)*
ALDS median (IQR)	77 (60-89)	78 (69-89)	65 (57-79)	76 (55-85)

Plus–minus values are means ± standard deviation. [a]Primary education: elementary or primary school. Secondary education: pre-vocational, senior general or pre-university. Higher education: higher professional or university. [b]Measured with numeric rating scale 0 (no fear) – 10 (maximal fear). SPPB = short physical performance battery, MMSE = minimal mental state evaluation, LVEF= left ventricle ejection fraction, ALDS = Amsterdam linear disability scale, and IQR = interquartile range. * P<0.05 values indicate significant differences between complete and missing data within each group (either intervention or control).

Supplementary Table 2. Regression results overview complete cases, complete follow up with imputed baseline values and intention to treat with multiple imputation

Outcome	Analysis Type	B (95% CI)	*p*-value
**SPPB-total score**	Complete cases	0.90 (0.14 to 1.64)	.020*
	Complete Follow Up + Imputation baseline	0.58 (–0.18 to 1.34)	.135
	Intention-to-treat + multiple imputation	0.56 (–0.31 to 1.43)	.202
**ALDS score**	Complete case	–1.77 (–6.07 to 2.53)	.417
	Complete Follow Up + Imputation baseline	–1.77 (–6.07 to 2.53)	.417
	Intention-to-treat + multiple imputation	–1.14 (–5.94 to 3.65)	.635
**Grip strength (kg)**	Complete case	–0.50 (–2.83 to 1.82)	.669
	Complete Follow Up + Imputation baseline	–0.50 (–2.83 to 1.82)	.669
	Intention-to-treat + multiple imputation	–0.14 (–2.26 to 1.97)	.893
**2MST steps**	Complete case	–0.79 (–9.45 to 7.88)	.858
	Complete Follow Up + Imputation baseline	–0.79 (–9.45 to 7.88)	.858
	Intention-to-treat + multiple imputation	–1.75 (–10.70 to 7.20)	.696

Complete cases (n=131), Complete follow-up with imputed baseline values (n=170), Intention to treat + multiple imputation (n=306); Abbreviations: B = Beta, 95% CI = 95% Confidence Interval, SPPB = Short Physical Performance Battery, ALDS = Amsterdam Linear Disability Scale, 2MST = Two Minute Step Test, kg = kilograms, * p-value <0.05.
